# Chronic non-bacterial osteomyelitis in children- five-year standardized follow-up of a prospective observational cohort in the pre-biological era

**DOI:** 10.1186/s12969-025-01106-2

**Published:** 2025-05-13

**Authors:** Christine Hofmann, Annette Holl-Wieden, Christiane Reiser, Meinrad Beer, Peter Raab, Henner Morbach, Hermann J. Girschick

**Affiliations:** 1 Pediatric Rheumatology, Immunology, Osteology, Children’s Hospital, University of Wuerzburg, Wuerzburg, Germany; 2 Clinic of Pediatric Rheumatology and Rare diseases, Children’s Hospital, University of Tuebingen, Tuebingen, Germany; 3 Department of Pediatrics, LKH Bregenz, Bregenz, Austria; 4 Department of Pediatrics, Division of Pediatric Rheumatology and autoinflammation reference center Tuebingen (arcT), Tuebingen, Germany; 5 Department of Radiology, University of Ulm, Ulm, Germany; 6 Section of Pediatric Orthopedics, University of Wuerzburg, Wuerzburg, Germany; 7 Children’s Hospital, Vivantes Clinic, Berlin-Friedrichshain, Berlin, Germany; 8 German Center for Growth and Development, DeuzWeg, Berlin, Germany

**Keywords:** CNO, CRMO, Osteomyelitis, Osteitis, Controlled follow-up, Composite disease activity measures, PedCNO, Outcome, Inactive disease, Remission

## Abstract

**Background:**

This prospective, long-term observational study, initiated in 2002, aimed to characterize clinical and laboratory data, whole body MRI detected lesions, and treatment responses in 37 juvenile patients with chronic non-bacterial osteomyelitis at a time when biological DMARDs were not yet standard therapy.

**Methods:**

Patients were assessed at baseline and at 1 (without MRI), 3, 6, 12, 18, 24, 36, 48, 60 months. All patients received naproxen as first-line therapy. Clinical management allowed for escalation to sulfasalazine, pamidronate, and glucocorticoids as needed. Treatment response was evaluated using the pedCNO disease activity score (30/50/70/90% improvement). Further composite numeric disease activity (DA) scores– the CARRA CDAS and a new MRI DAS - were applied.

**Results:**

The mean age at disease onset was 10.8 years, with a diagnostic delay of 5.8 months. Naproxen was the initial treatment in all patients. Second-line therapy was initiated in 10 patients due to inadequate improvement in physician global assessment of disease activity, patient-reported overall wellbeing or MRI lesions. Escalated therapies included sulfasalazine (*n* = 10), bisphosphonates (*n* = 1), methotrexate (*n* = 1), and short- (< 4 wks) or long-term oral glucocorticoids (*n* = 5 and *n* = 3, respectively). The mean number of clinical lesions decreased from 2.1 to 0.4 at 12 months and reached 0.15 at 60 months. MRI-detected lesions declined from 5.0 to 2.25 at 12 months and to 1.1 at 60 months.

**Conclusion:**

Most children experienced favourable long-term outcomes. Clinical improvement occurred more rapidly than radiologic resolution. Patients with insufficient response to NSAIDs should be considered for a treat-to-target approach, including the use of conventional and biologic DMARDs.

**Trial registration:**

A trial registration EUDRA CT was not available at the time the study was started. Informed consent was given by all parents.

**Supplementary Information:**

The online version contains supplementary material available at 10.1186/s12969-025-01106-2.

## Background

Chronic non-bacterial osteomyelitis (CNO) has been recognized in children for over five decades [1, 2]. While CNO refers to the overall spectrum of this autoinflammatory disease [3], the term chronic recurrent multifocal osteomyelitis/osteitis (CRMO) is often used to describe its more severe, relapsing form [4, 5]. CNO is classified among autoinflammatory diseases. Despite an incomplete understanding of its pathogenesis, this assumption is supported by observations of dysregulated myeloid cells and NLRP3 inflammasome activation [6–13]. Histopathology typically reveals both acute and chronic inflammation with features of bone remodelling, although lesion variability can complicate both diagnosis and the assessment of disease activity (DA) [5, 14, 15].

Nonsteroidal anti-inflammatory drugs (NSAIDs) are considered first-line therapy and have shown efficacy in many patients [15–23]. For those with more persistent or severe disease, treatment options include glucocorticoids [22, 24], conventional synthetic disease modifying anti rheumatic drugs (csDMARDs) such as sulfasalazine and methotrexate, interferon-alpha, bisphosphonates [6, 25–27] and biological DMARDS (bDMARDs) such as tumor necrosis factor (TNF) α-inhibitors [[16, [28, [29] and more recently, IL-1 blockade [11].

To facilitate standardized treatment and assessment, consensus treatment plans (CTP) and treat-to-target (T2T) strategies have been proposed [30]. However, discrepancies between histological findings, its magnetic resonance imaging (MRI) characteristics, and patient- or parent-reported symptoms complicate objective DA evaluation. Several single or composite DA scores have been developed to address this challenge, including parameters such as clinical or MRI-detected number of lesions [30], physician global assessment of DA (PGDA) or patient/parents global assessment [31]. Among these, the pedCNO score (31) and the numeric composite, patient focused Clinical DA score (CDAS) by the Childhood arthritis and Rheumatology Research Alliance (CARRA) were proposed [32]. Nevertheless, widely accepted definitions for CNO DA, response, inactive disease and remission however remain elusive [12, 30–33], and there is a clear need for validated tools for use in both trials and practice [34, 35]. Current understanding of CNO is largely based on retrospective cohort analysis [36–44], with a notable scarcity of long-term prospective data [32, 45].

To address this gap, we conducted a prospective, standardized 5-year evaluation of DA in a pediatric CNO cohort. Our primary treatment objective was complete resolution of DA, defined as a lesion-free whole body MRI (WB-MRI). We assessed clinical, patient-/parent-reported, and radiological parameters using several composite DA scores to explore their utility in a treat to target framework.

## Methods

### Statement of ethics and consent

The study was approved by the ethics committee of the University of Wuerzburg (68/2003) and was performed according to the declaration of Helsinki. A trial registration EUDRA CT was not available at that time. Informed consent was given by all parents. The datasets used and analysed during the current study are available from the corresponding author on reasonable request.

### Description of the patient cohort

A total of 37 children (24 girls, 13 boys) newly diagnosed with CNO were included in the study. All patients were treatment-naïve at the time of inclusion, having received neither anti-inflammatory nor antibiotic therapy. The disease was assessed by initial diagnostic biopsy, laboratory tests and serial imaging, including WB-MRI at baseline and at 3, 6 and 12, 18, 24, 36, 48, 60 months. The PedCNO core set of outcome variables comprised five measures: (1) ESR, (2) number of radiological lesions (WB-MRI), (3) physician’s global assessment of DA, (4) patient or parent global assessment, and (5) CHAQ. Improvement from baseline was defined according to the PedCNO response criteria: PedCNO30, PedCNO50, PedCNO70, and Ped CNO90, representing at least 30%, 50%, 70%, and 90% improvement, respectively, in at least three out of five core variables, with no more than one of the remaining variables worsening by more than at the same percentage threshold. All patients initiated treatment with naproxen 15 mg/kg/day at the time of diagnosis or biopsy. In case of insufficient response, sulfasalzine was introduced as a DMARD (For further details, see supplemental Table 1). The treatment goal was complete resolution of radiological lesions as determined by WB-MRI. Shared decision-making between families and treating physicians guided the initiation of DMARDs and glucocorticoid usage. Initial one-year initial follow-up for this cohort has been previously reported [46].

### Assessment of DA

To assess the DA, an in-depth physical examination for the definition of clinically (patient) noted lesions (site, number), Erythrocyte sedimentation rate (ESR) was performed.

### Single numeric and composite rating scores for disease activity

WB-MRI scans were evaluated by an independent pediatric radiologist with expertise in CNO (MB), who was blinded to the patient’s clinical status [47, 48]. Disease burden was quantified by determining the number of inflammatory lesions. DA was further assessed using numerical rating scales (NRS, scale 0–10, with 10 indicating highest severity) for the following parameters: patient/parent (PAG) and physician estimated global disease activity (PGDA), and patient-reported pain (PAP). Functional impairment was evaluated using the child version of the HAQ (C-HAQ) [31]. These parameters were integrated into the composite DA score, pedCNO, which defined response thresholds of 30%, 50%, 70%, and 90% improvement relative to baseline [31]. The score incorporates NRS assessment of parent/patient and physician estimated DA, parent/patient pain rating, the C-HAQ score, and the number of WB-MRI-defined lesions. As the pedCNO score is designed to measure change from baseline rather than absolute disease burden [46], further refinement of scoring appeared justified. The goal was to establish easily comparable numeric metrics over time. Notably, pedCNO does not capture baseline disease activity itself - an important limitation given the need to characterize disease severity at study entry [35].

To better capture patient-centred outcomes, we calculated the recently proposed „clinical“ DA score (CDAS), defined by CARRA as a composite of PAG/PAP/Clin (patient global DA, patient pain, clinically parents/patient noted lesions) [32]. We had previously described that patient-reported outcomes, especially pain, may rapidly improve with treatment and may no longer show responsiveness after several months of CNO treatment [31]. To address this limitation, we developed an adapted DA score incorporating objective imaging findings– specifically, the number of active lesions on WB-MRI– alongside patient-reported measures. This MRI-based score (MRI DAS PAG/PAP/MRI) provides a more stable and quantifiable assessment of disease burden over time. Recognizing the values of clinical expertise, we further proposed and additional composite score incorporating PGDA: MRI DAS PGDA/PAG/MRI. For comparative purposes, a purely clinical score (DAS PGDA/PAG/Clin) combining PGDA, PAG, and clinically observed lesions, was also evaluated. Notably, scores that substituted patient pain for patient global assessment performed similarly in terms of absolute values (see supplemental Table 2). The use of standardized numeric DA scores may facilitate meaningful longitudinal comparisons within individual patients and across cohorts.

Relapse/Flare was defined as the recurrence of pain and local swelling, accompanied by MRI-confirmed signs of inflammation on fluid sensitive sequences. Inactive disease was defined by the absence of clinical symptoms (pain, local swelling, functional impairment in adjacent joints (clinical lesion count = 0), PGDA = 0, PAG = 0, C-HAQ = 0, and achievement of ≥ 90% improvement in the PedCNO composite score [34]. In addition, no MRI evidence of active inflammation (i.e., no lesions) was required. The patient’s treatment status (on or off medication) was documented. Remission was defined as sustained inactive disease for at least 12 consecutive months without medication and no documented flares or relapses during the follow-up period.

### Statistical analysis

Standard descriptive statistics were used to describe the distribution of sociodemographic, clinical, laboratory and imaging parameters (Graph Pad Prism 10.1.2.). T-Test (two-sided, unpaired; using Excel) was performed to compare the individual slopes for each patient (CARRA CDAS PAG, PAP Clin versus MRI DAS PAG PAP, MRI lesions) in the study period of 3–18 months. For multiple comparisons we used a mixed effects analysis (REML) followed by a Tukey`s multiple comparison test. P values less than 0.05 were considered significant.

## Results

### Therapeutic strategy according to the study protocol

All 37 patients received NSAID therapy with naproxen (15 mg/kg/day), with a mean treatment duration of 29.1 months. Ten required escalation to sulfasalazine (30–50 mg/kg body weight (BW)/day divided in 2 to 3 single doses/day), initiated at a mean of 16.6 months after diagnosis and continued for an average of 33.7 months. Additionally, 8 of 37 patients received adjunctive glucocorticoids alongside sulfasalazine. Glucocorticoid treatment was started with prednisolone at 2 mg/kg BW/day for one week, followed by tapering during the second week. If ongoing treatment was required, a maintenance dose of 0.2 mg/kg BW/day was administered. The cumulative duration of glucocorticoid therapy at or below 0.2 mg prednisolone/kg/day, averaged 17 months. Among these, three patients received long-term glucocorticoid treatment (12, 32, and 72 months), while five received therapy for less than two weeks. Notably, no cushingoid side effects were observed. Two patients required additional DMARD beyond sulfasalazine: one received bisphosphonates, and one patient with co-manifestation of inflammatory bowel disease was treated with methotrexate.

### Laboratory analysis

No statistically significant changes were observed in routine hematologic parameters, including leukocyte and platelet counts or hemoglobin levels, over the study period. In contrast, a significant decrease in erythrocyte sedimentation rate (ESR) was noted - from a baseline of 27 mm/h to 12 mm/h after 3 months - which remained low over time. These findings suggest that ESR may serve as a reliable marker of disease activity in the early phase of CNO management (Figure S1 supplement).

### DA measurement by clinical and MRI lesions

**Fig. 1:**
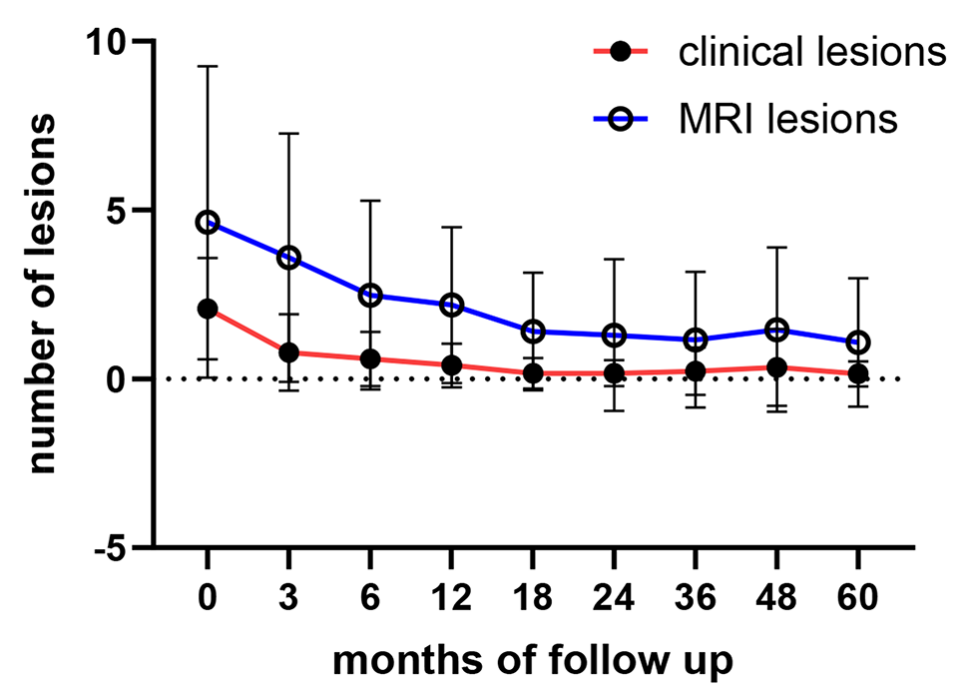
Clinical and whole body MRI lesions during five years of follow-up. The mean number of clinically noticed lesions by the patients/ parents, as well as the MRI detected lesions in whole body TIRM technique are shown over the study period of 5 years

At baseline, patients/parents reported a mean of 2.1 clinically detectable lesions per patient. However, WB-MRI revealed a higher burden, with an average of 5 lesions per patient. Naproxen treatment correlated with a notable clinical improvement: by 18 months, the mean number of clinically reported lesions per patient had decreased to 0.17, with slight increase to 0.34 observed at 48 months (Fig. 1). MRI-based assessment showed a steady decline in the number of lesions over the first three years of follow-up, reaching a nadir of 0.96 detectable lesions on average (Fig. 1). However, in the fourth year, the mean number of MR-detected lesions rose to 1.55, driven by disease flares in three patients. Adjustments in therapy according to the study protocol, including the addition of DMARDs, helped control these flares in two of these patients over the following 12 months. By the end of the follow-up, the mean number of clinically reported lesions was 0.15, and the mean number of MRI-detected lesions was 1.05.

### Clinical DA measurement using single numeric scores

**Fig. 2:**
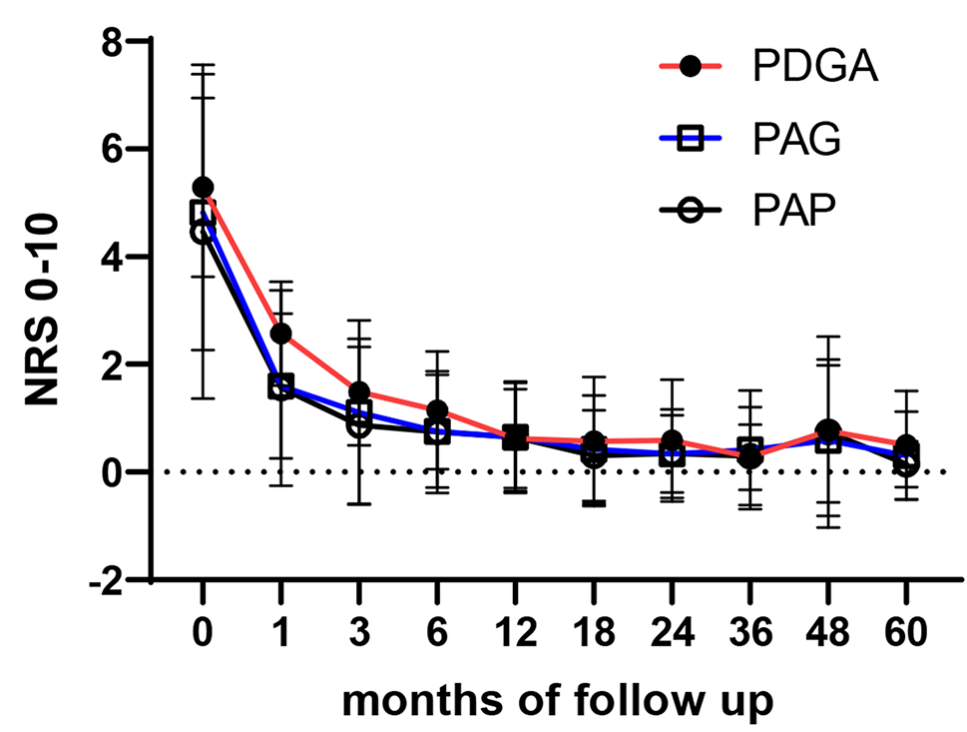
Single disease activity measures PGDA, PAG, PAP. Single disease activity measures including the physician defined global assessment of disease activity PGDA, the patient noticed global disease activity PAG, and the patient noticed pain PAP are depicted in means of numeric rating scales in our patients during follow-up

Patient/parent-reported overall well-being (PAG) and pain (PAP) scores decreased significantly within the first 6 months of therapy, decreasing from a baseline score of 5 to approximately 0.6 after five years. Similarly, physician’s rated global disease activity (PGDA) declined within the first 2 years, with a mean NRS decreasing from 5.3 to 0.6 within the first 12 months. Notably, PGDA values remained slightly higher than PAG and PAP scores during the first two years of follow-up (Fig. 2). Additionally, a significant decline in the C-HAQ score was noted within 3 to 6 months and reaching a score of 0, indicating no functional impairment (Figure S2 supplement). Disease flares at the fourth year mark were reflected in all three single DA measures (Fig. 2), as well as the C-HAQ, which rose modestly to 0.1 at 48 months (Figure S2 supplement).

### Clinical DA measurement using composite scores

#### The pediatric CNO score

**Fig. 3:**
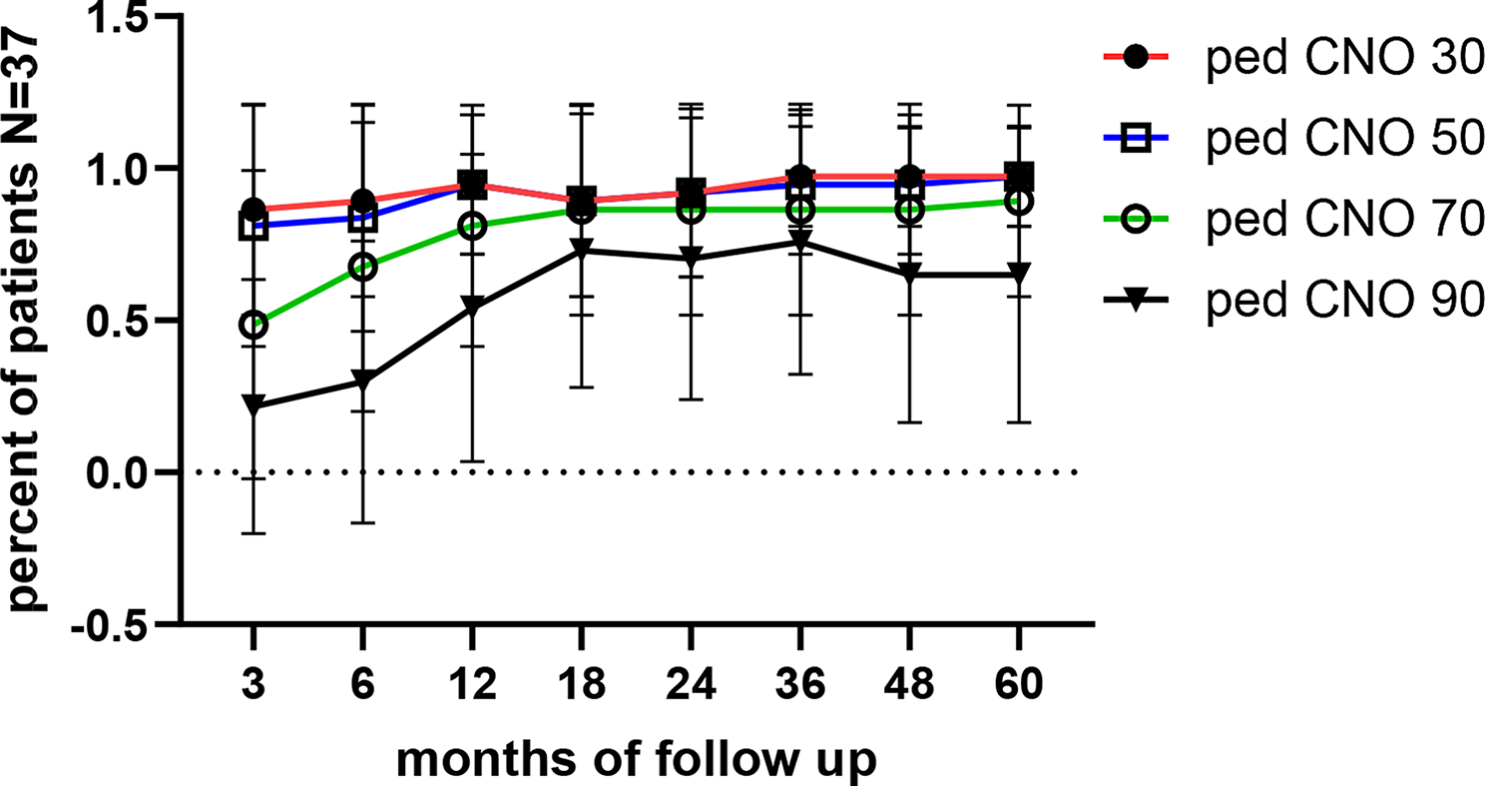
PedCNO score with 30, 50, 70, 90% of improvement categories. Improvement rates of the pediatric CNO disease activity composite score are depicted as the percent of patients reaching this improvement category

We previously reported within this cohort that over 80% of patients achieved at least 70% improvement in the pedCNO score after 12 months of treatment (Fig. 3) [46]. In this extended follow-up, we assessed 90% improvement levels: by 12 months, 54% of patients reached this threshold, rising to 70% at 24 months and 76% at 36 months (Fig. 3). However, flares occurring in year four led to a decline, with only 65% of patients maintaining this response at 48 and 60 months.

### Numeric composite DA scores

**Fig. 4:**
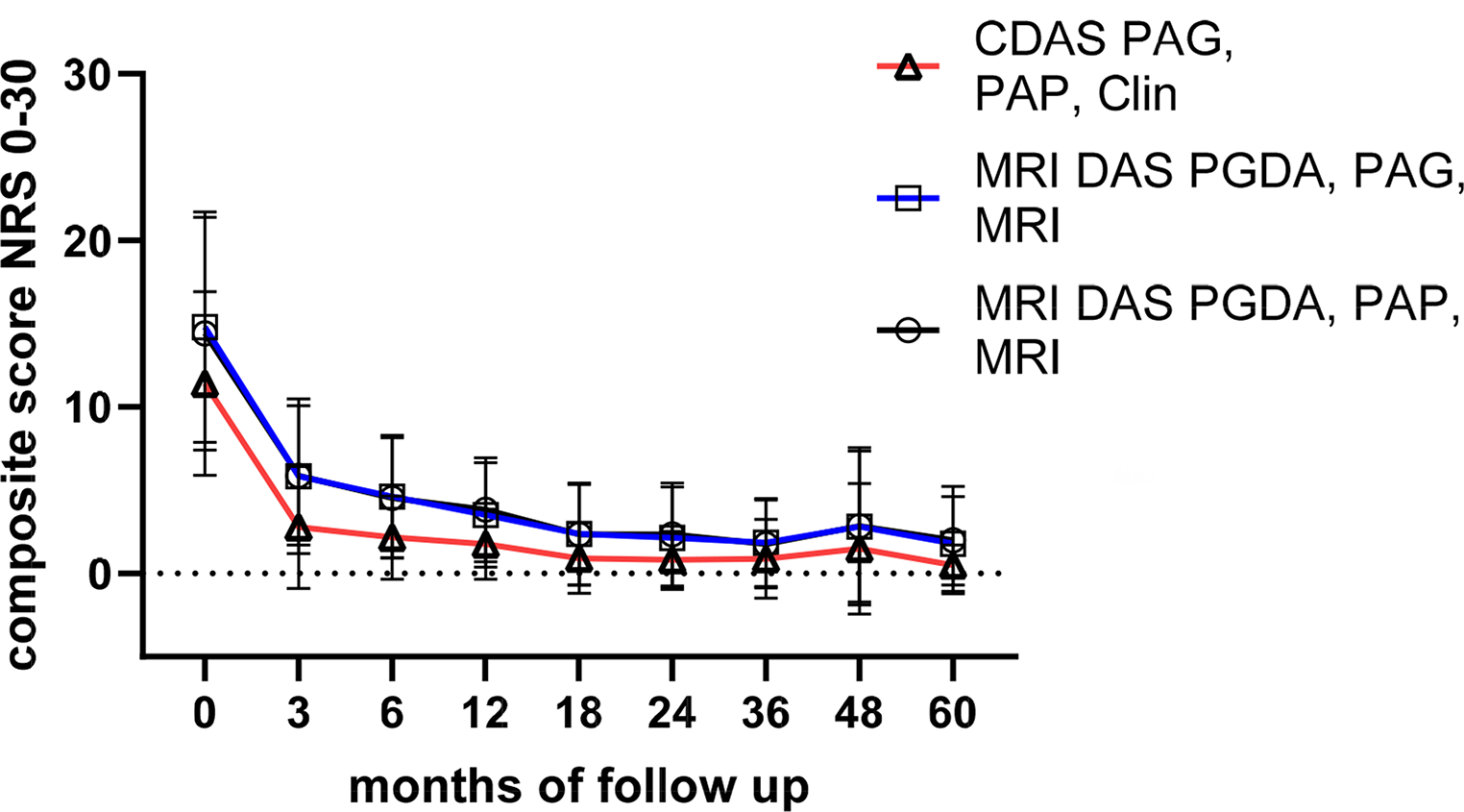
Time course of the patient centred CDAS score in comparison with physician/patient and MRI weighted disease activity scores. Composite scores based on numeric rating scales are given as mean of the patient cohort based on the summation off single disease activity measures PAG, PAP, PGDA, Clin (Clinical lesions) or MRI (Magnetic resonance imaging based on whole body TIRM technique)

Using the CDAS PAG/PAP/Clin, as proposed by CARRA [32], the mean score at baseline was 11.4, which declined rapidly to 2.1 after 12 months (Fig. 4, Figure S3). According to CARRA definitions, CDAS PAG/PAP/Clin score values below 2.5 indicate “inactive disease”, 2.5–7.5 suggest “mildly active disease”, 7.5–12.5 “moderately active CNO disease”, and > 12.5 “severely active disease” [32].

We further explored variations of the DAS by incorporating the PGDA or MRI findings. Scores including PGDA (DAS PGDA/PAG/Clin, DAS PGDA/PAP/Clin) were consistently higher than patient-reported scores during the first 36 months due to generally higher physician rating (Figure S3).

As expected, replacing clinically noted lesions with whole-body **MRI**-defined lesions yielded higher scores [34, 46] (MRI DAS PAG/PAP/MRI, MRI DAS PGDA/PAG/MRI, MRI DAS PGDA/PAP/MRI). These three MRI DA measures (Figure S4), as well as the three clinically lesion-based DA scores (Figure S3) reflected disease activity over time, including flares. During the first year, patient-reported scores declined more steeply (Fig. 4). By the second year, these different scores began to converge, showing comparable values by year three.

Analyzing curve slopes, the CARRA CDAS rapidly decreased by 75.4% in the first three months from 11.4 to 2.8, and plateaued further with a mean decline of 0.057 NRS points per month (Fig. 4). Conversely, the MRI-based DAS, including PGDA, dropped from 14.4/14.8 to 5.8 (60% reduction) after 3 months, followed by a decrease of 0.13 NRS per month until month 36 (see Fig. 4). The differences in slope between CDAS and MRI DAS PAG PAP, MRI lesions between month 3 to 18 was significantly different (*p* = 0.042; two sided t-test).

In one patient with suspected pain amplification syndrome, no MRI lesions were observed after 24 months. However, both PAG and PAP remained elevated further (NRS around 4), while PGDA ranged from 0 to 2. The overall pedCNO score showed 90% improvement at 36 and 48 months, and 50% at 60 months– despite persistent patient-reported symptoms. In contrast, the CDAS PAG/PAP/Clin (9.2, 1.4, 6), MRI DAS PAG/PAP/MRI (9.2, 1.4, 6), and MRI DAS PGDA/PAG/MRI (4, 0.5, 6), as well as the PGDA (0, 0, 2) remained elevated after 36, 48, and 60 months, respectively, reflecting the clinical disconnect.

Conversely, another patient exhibited accumulating silent MRI lesions: MRI lesions increased from 2 at baseline to 9 at 48 months and 8 at 60 months. Despite this, PAG dropped to 0 after 12 months and remained at 0 with only slight increase at 18 months (1.0) and 60 months (0.5). PAP scores remained low throughout (0.6, 0.3, 0.2, 0.5 at 24, 36, 48, and 60 months, respectively). PGDA scores ranged from 1 to 4 during follow-up, indicating higher physician concern. CDAS PAG/PAP/Clin remained < = 1 after 24 months, while MRI DAS PAG/PAP/MRI (values 6, 3, 8, 9) tracked with lesion progression. A short 2-week glucocorticoid course at 12 months and sulfasalazine initiation at 18 months stabilized disease.

### Therapeutic outcomes in patients requiring DMARDs

Of the ten patients treated with DMARDs, three finally achieved a pedCNO90, seven reached a pedCNO70, and nine attained a pedCNO50. One patient failed to meet pedCNO improvement criteria, although disease activity remained stable. This patient’s PGDA decreased from 3.0 to 0.5, PAG from 2.0 to zero and MRI lesions increased from one to two without further progression. CDAS PAG/PAP/Clin decreased from 8.0 to 0.2 and MRI DAS PAG/PAP/MRI from 8.0 to 2.2 and MRI DAS PGDA/PAG/MRI from 6 to 2.5 at 60 months. Despite moderate and stable disease activity, treatment with sulfasalazine and low-dose glucocorticoids did not achieve inactive disease or remission. Initially, pain (PAP) dominated DA scores; later, persisting MRI lesions accounted for most of the activity in this patient. The patient declined escalation to bisphosphonates. Physicians rated the treatment response as suboptimal based on imaging.

### Inactive disease

**Fig. 5:**
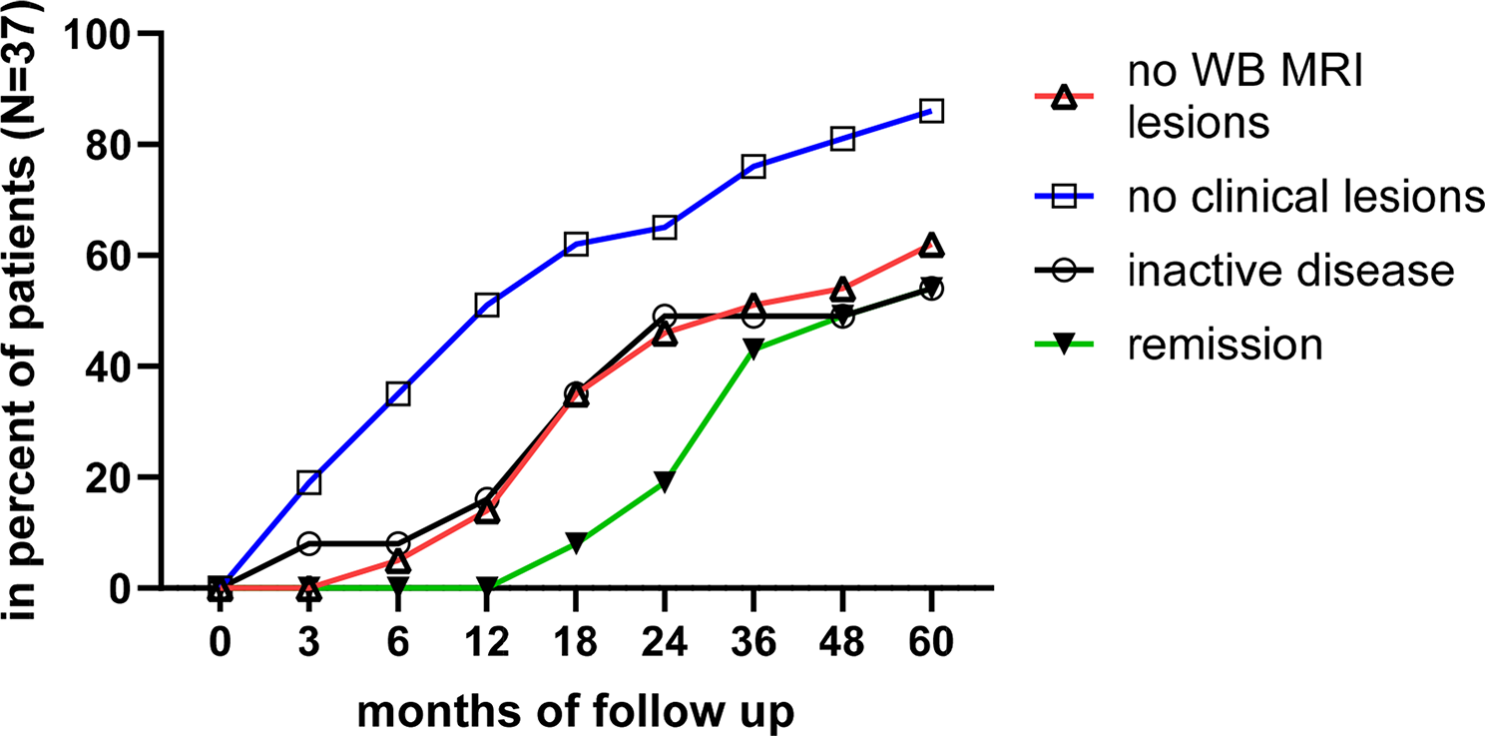
Inactive disease and proposal of remission in CNO. The status of no clinically noted lesions in addition to no whole body MRI lesions is depicted in the percent of patients throughout the study. Composite inactivity measures inactive disease as well as patients reaching remission (inactive disease without medication since 12 months) are outlined

Based solely on the absence of clinical lesions, nearly 90% of patients achieved inactive disease (IA) status at 60 months (Fig. 5). MRI-confirmed absence of lesions was observed in approximately two-thirds of patients by the end of the follow-up (Fig. 5). We applied a strict composite IA definition requiring: absence of clinical and MRI lesions, PGDA = 0, PAG = 0, C-HAQ = 0, and a pedCNO of 90%. Using this definition, 16% of patients achieved IA at 12 months, increasing to 49% at 24 months and 54% at 60 months. This IA definition holds important clinical relevance, though it remains open to further discussion. In parallel, the newly proposed CDAS PAG/PAP/Clin defines a composite NRS below 2.5 as indicative for inactive disease (32). Applying this threshold, 24 out of 37 patients (65%) met the criterion for inactive disease after three months in this study. Notably, two of these patients demonstrated complete resolution of MRI-detected lesions at that time point.

To refine **remission**, we required sustained inactive disease - as per the composite IA definition– for at least 12 consecutive months, combined with discontinuation of all anti-inflammatory medications during that period. Using this strict definition, approximately 50% of patients achieved remission at 48 months, increasing slightly to 54% at 60 months. At the five-year mark, 60% of patients showed no MRI lesions, and 86% were clinically symptom-free (Fig. 5).

### Height over time

A notable decline in personal height was observed in the cohort after 2.5 years of follow-up. At the end of the study period, 8% of CNO patients (*n* = 3) had a height below the 2nd percentile when compared to national reference standards (Figure S5 in the supplement). Among these patients, one had a co-manifestation of inflammatory bowel disease, while another carried a heterozygous variant of unknown significance in the *IL1RN* gene. The third patient, a female, showed an otherwise favorable response without additional clinical features suggesting short stature by other means.

## Discussion

Prior to this study, many clinicians treated CNO based on patient-reported symptoms, using on-demand anti-inflammatory or analgesic strategies [5, 12]. This often led to persistent disease activity and long-term damage [49]. In response, our study– initiated in 2002– implemented a treat-to-target (T2T) approach aimed at achieving a MRI-confirmed disease-free state. We report long-term prospective outcome in 37 CNO patients, evaluating the effectiveness of naproxen, sulfasalazine, and oral glucocorticoids.

Multiple DA measures were assessed, including the composite pedCNO score [46], the CARRA CDAS and patient, physician and MRI-based assessments including also measures for inactive disease and remission [34]. To achieve the study target of an “MRI disease-free state,” treatment with naproxen was continued beyond clinical resolution of symptoms. Notably, patient- and physician-reported global assessments often indicated inactive DA or mild disease within few months, whereas MRI continued to reveal “active” lesions [31, 34]. This raises question about the adequacy of the CARRA proposed CDAS DA categories of inactive disease (CNO CDAS score values < 2.4), mildly active disease (2.5–7.4), moderately active disease (7.5–12.4), and severely active disease (> 12.5) in reflecting true disease burden [32]?

Although continued treatment based on imaging may carry the risk of overtreatment, relying solely on clinical assessments risks premature discontinuation and potential chronic disease progression.

Indeed, our treatment algorithm led to significant clinical and imaging improvements over time [46] Compared to retrospective international registries, our IA rates aligned with or surpassed reported results [40, 50]. We previously considered NRS rating scales of PGDA below 1 as indicative of inactive disease in CNO [31, 34]. In this study, we applied an even more stringent definition - requiring sustained inactive disease for one year without medication to define remission. A significant proportion of patients achieved this disease-free state by year five.

Our data underscore the limitations of relying on single metrics. Patient-reported scores declined quickly in the first months, likely underestimating subclinical inflammation. MRI often detected silent lesions, which were not perceived by patients, emphasizing the complementary value of imaging and physician assessments [34, 46]. Conversely, the clinical relevance of MRI-detected but asymptomatic lesions remains uncertain, and pain amplification syndromes must be considered when interpreting symptoms in the absence of imaging findings.

The pedCNO score demonstrated robust responsiveness, incorporating both patient and physician perspectives, as well as imaging data. Although the C-HAQ’s relevance in CNO is debated, it showed good correlation with early clinical improvements [46]. While the requirement for complete disease inactivity and 12-month remission may be too stringent for broader registry use, our results suggest these targets are achievable in a subset of patients even without access to biologics.

In today’s clinical landscape, patients unresponsive to NSAIDs or csDMARDs would likely receive biological therapy. In our cohort, five patients would have met criteria for escalation based on MRI lesion counts despite clinical improvement. Modern T2T strategies should incorporate multi-dimensional assessments—including MRI, PGDA, and patient input—to guide individualized decisions on treatment escalation, such as TNF inhibitors or bisphosphonates in refractory cases.

## Conclusion

This long-term, prospective cohort study provided a comprehensive evaluation of DA and treatment efficacy in pediatric CNO, using a targeted treatment strategy focused on achieving MRI-confirmed remission. The findings offer valuable insight into pre-biologic managements and may serve as a historical reference for future T2T trials.

These data are relevant not only for pediatric CNO, but also for adult CNO or SAPHO syndrome, informing treatment decisions in the evolving era of targeted therapies.

## Electronic supplementary material

Below is the link to the electronic supplementary material.

Supplementary Material 1

## Data Availability

No datasets were generated or analysed during the current study.

## Abbreviations

BW: Body weight
CARRA: Childhood arthritis and Rheumatology Research Alliance
CDAS: Clinical disease activity score
C-HAQ: Childhood health assessment questionnaire
Clin: Patients’ assessment of the number of clinical lesions
CNO: Chronic non-bacterial osteomyelitis
csDMARD: conventional synthetic disease modifying anti rheumatic drug
CRMO: Chronic recurrent multifocal osteomyelitis
CTP: Consensus treatment plans
DA: Disease activity
DMARD: Disease modifying anti rheumatic drug
IA: Inactive disease
ESR: Erythrocyte sedimentation rate
MRI: Magnetic resonance imaging
NPRD: GERMAN national pediatric rheumatologic database
NSAID: Non-steroidal anti-inflammatory drugs
PAG: Patients’ global disease activity assessment
PAP: Patients’ pain assessment
pedCNO: pediatric CNO disease activity score
PGDA: Physician definded global assessment of disease activity
SAPHO: Synovitis, acne, pustulosis, hyperostosis, osteitis/osteomyelitis
TIRM: TURBO inversion recovery magnitude resonance MR imaging
T2T: Treat to target treatment guidelines
TNF: Tumor necrosis factor
