# Supplementary material for: Chronic non-bacterial osteomyelitis in children- five-year standardized follow-up of a prospective observational cohort in the pre-biological era
Source: Pediatr Rheumatol Online J. 2025 May 13;23:50. doi: 10.1186/s12969-025-01106-2 (PMC12076821; doi:10.1186/s12969-025-01106-2)
Supplement: Supplementary file 1 — Supplementary Material 1 [file 12969_2025_1106_MOESM1_ESM.docx]

**Supplemental methods:**

**Set up of the Study and Description of the cohort**

We prospectively included juvenile patients with newly diagnosed CNO in a community-based academic pediatric rheumatology referral clinic. Diagnosis of CNO was confirmed by clinical signs of osteomyelitis (pain, local swelling, impairment of limb motion). Diagnostic procedures including X-rays and WB-MRI in all patients. All patients were biopsied and had not received antibiotic or anti-inflammatory medication before. Between 2002 and 2007 37 children with CNO were included into the study (mean age 10.3 years at onset of symptoms, range 2-16 years, 24 girls, 13 boys). Patients were followed at controlled time points up to 5 years, in detail after 1, 3, 6, 12, 18, 24, 36, 48 and 60 months. Clinical, laboratory and radiological data were reported at disease onset and up to 60 months. The one-year initial follow-up of this cohort was reported before([46](#_ENREF_46)). Laboratory tests included blood count, ESR, CRP, serum ferritin, serum IgG, IgM, IgA, and HLA B27. WB-MRI was performed after 3, 6, 12, 18, 24, 36, 48 and 60 months. Standard X-rays were not routinely performed during follow-up. Initial diagnostic bone biopsy included extensive microbial workup in all patients. Standard culture techniques to detect fungi, mycobacteria, aerobic and anaerobic bacteria in all samples and eubacterial polymerase chain reaction for molecular detection of bacterial ribosomal DNA were performed([15](#_ENREF_15)).

**Table S1 supplement: Patients' clinical features at inclusion and therapy during follow-up**

**Table 2 supplement: Single and composite numeric disease activity measures**

| **single numeric disease activity measures** | | | | | | |  |  |
| --- | --- | --- | --- | --- | --- | --- | --- | --- |
|  | | PAG | | patients’ global disease activity assessment | |  |  |  |
|  | | PAP | | patients’ pain assessment | |  |  |  |
|  | | PGDA | | physician defined global assessment of disease activity | |  |  |  |
| **composite numeric disease activity measures** | | | | | | | | |
| cDAS CARRA | | PAG/PAP/Clin | |  | | |  |  |
| DAS | | PGDA/PAG/Clin | |  | | |  |  |
| DAS | | PGDA/PAP/Clin | |  | | |  |  |
|  | | |  | |  | | |  |
| MRI DAS | | PAG/PAP/MRI | |  | | |  |  |
| MRI DAS | | PGDA/PAG/MRI | |  | | |  |  |
| MRI DAS | | PGDA/PAP/MRI | |  | | |  |  |

**Figure S1 supplement: ESR during follow-up.** The mean of erythrocyte sedimentation rate (ESR) of all patients is given, as well as the standard deviation throughout the study time.

**Figure 2 supplement: C-HAQ during follow-up.** The mean C-HAQ is shown over the study period of 5 years.

**Figure S2 supplement: Composite disease activity scores including the clinically noticed number of lesions by the patients/parents.** Composite scores based on numeric rating scales are given as mean of the patient cohort based on the summation off single disease activity measures PAG, PAP, PGDA, Clin.

**Figure S3 supplement: Composite disease activity scores including the whole body MRI detected number of lesions.** Composite scores based on numeric rating scales are given as mean of the patient cohort based on the summation off single disease activity measures PAG, PAP, PGDA, MRI (Magnetic resonance imaging based on whole body TIRM technique)

**Figure S4 supplement: Height during follow-up.** The mean of standard deviations of height is shown in relationship to a German age and sex related reference cohort during follow-up.

Fig. Supp.2

Fig. Supp. 1

Fig. Supp.3

Fig. Supp.4

Fig. Supp.5
